# The association between lipoprotein(a) and atrial fibrillation: A systemic review and meta‐analysis

**DOI:** 10.1002/clc.24086

**Published:** 2023-07-12

**Authors:** Mingyang Yang, Basma Nasr, Junzhao Liu, Yu Du, Jiayin Yang

**Affiliations:** ^1^ Department of Liver Surgery, Liver Transplantation Center West China Hospital of Sichuan University Chengdu Sichuan China; ^2^ West China Fourth Hospital/West China School of Public Health Sichuan University Chengdu Sichuan China; ^3^ Department of Cardiology First Affiliated Hospital of Dalian Medical University Dalian Liaoning China; ^4^ Health Emergency Management Research Center, China‐PUMC C.C. Chen Institute of Health Sichuan University Chengdu Sichuan China; ^5^ Department of Emergency and Critical Care Medicine, West China School of Public Health, West China Fourth Hospital Sichuan University Chengdu Sichuan China

**Keywords:** atrial fibrillation, lipoprotein(a), meta‐analysis

## Abstract

Lipoprotein(a) (Lp[a]) is a particle consisting of a low‐density lipoprotein (LDL)‐like core connected to an apolipoprotein(a) chain, which is an established risk factor for cardiovascular disease. However, studies addressing the relationship between atrial fibrillation (AF) and Lp(a) demonstrated conflicted results. Thus, we sought to evaluate this relationship by conducting this systemic review and meta‐analysis. We performed a comprehensive systematic search of health science databases, including PubMed, Embase, Cochrane Library, Web of Science, MEDLINE, and ScienceDirect, to identify all relevant literature from their inception to March 1, 2023. We identified nine related articles, which were eventually included in this study. Our study showed no association between Lp(a) with new‐onset AF (HR = 1.45, 95% confidence interval [CI]: 0.57–3.67, *p* = .432). In addition, genetically elevated Lp(a) was not associated with the risk of atrial fibrillation (OR = 1.00, 95% CI: 1.00–1.00, *p* = .461). Different stratification of Lp(a) levels may have different outcomes. Also, higher Lp(a) levels may be inversely associated with the risk of developing AF compared to those with lower levels. Lp(a) levels were not associated with incident AF. Further research is needed to identify the mechanism underlying these results and better understand Lp(a) stratification for AF and the possible inverse association between Lp(a) and AF.

## Graphical abstract

A systemic review and meta‐analysis evaluating the association between Lipoprotein(a) (Lp[a]) and atrial fibrillation. The final results showed no association between Lp(a) levels and incident atrial fibrillation.

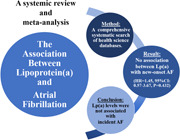

## 1 INTRODUCTION

Atrial fibrillation (AF) is the most common type of cardiac arrhythmia, characterized by rapid and irregular activation of the atria.^1^, ^2^ The global prevalence of AF risk factors is significantly increasing, raising AF prevalence and complications, such as Ischemic stroke, heart failure, and death, creating significant public health burdens worldwide.^3^, ^4^ Early detection and prevention of AF will significantly impact the disease's management and prognosis before any structural and electrical remodeling occurs in the atria, leading to further remodeling that will exacerbate the condition.^5^, ^6^

Lipoprotein(a) (Lp[a]) is a particle consisting of a low‐density lipoprotein (LDL)‐like core connected to an apolipoprotein(a) chain.^7^, ^8^ Lp(a) is an established risk factor for cardiovascular diseases such as coronary artery disease, ischemic stroke, and aortic valve stenosis.^9^, ^10^, ^11^ Studies suggested mechanisms promoting such effect are through Lp(a) pro‐atherosclerotic acting as a lipoprotein and its prothrombotic properties via its similarity to plasminogen. Rehberger Likozar and colleagues suggested that Lp(a) contributes to the development of atherosclerosis in several ways, including increasing the expression of vascular cell adhesion molecule‐1 and E‐selectin in endothelial cells; enhancing the accumulation of peripheral blood mononuclear cells in vessels; acting as a pro‐inflammatory through its oxidized phospholipids.^12^ On the other hand, Lp(a) also has adverse effects on the cardiovascular system outside of atherosclerosis and atherothrombosis, such as calcific aortic sclerosis.^9^

However, the association between Lp(a) and AF is still exploratory. Although Lp(a) is a recognized risk factor for coronary artery disease, and coronary artery disease is a risk factor for AF, it is suggested that Lp(a) particles have additional thrombogenic and inflammatory properties that could provide other mechanisms to regulate AF.^13^ Unlike coronary artery disease, ischemic stroke, and aortic valve stenosis, an effect of Lp(a) on AF has been suggested but has not been effectively evaluated.^14^ Thus, the relationship between Lp(a) and atrial fibrillation (AF) is uncertain.

Multiple studies have successfully proven the significant role of Lp(a) in the development of cardiovascular diseases such as coronary heart disease.^9^, ^15^ One study suggested a significant association with a risk ratio after adjustment for age and sex.^15^ Conversely, studies discussing Lp(a) role in developing AF are scarce. It is proven that Lp(a) levels are associated with atherosclerotic cardiovascular disease, which, in turn, is associated with incident AF.^16^, ^17^ However, to this date, prospective Lp(a) levels associations with incident AF have not been thoroughly investigated.

A case–control study in Spain showed that Lp(a) levels were not associated with AF.^18^ Another cohort study that included 79 patients has shown that Lp(a) levels were not associated with AF recurrence after electrical cardioversion in 2 years of follow‐up.^19^ On the other hand, a multivariable Mendelian randomization study suggested a positive causal association between high Lp(a) levels and the increased risk of AF.^20^ Recent mechanistic studies suggest a potentially active role for cholesterol in preventing AF, possibly through specific lipid‐stabilizing effects on cell membranes.^21^ Another study has suggested an inverse relationship where low levels of both apolipoprotein A1 (ApoA1) and B (ApoB) were associated with incident AF.^22^ A cohort study conducted in China showed that TC and LDL‐C levels were inversely associated with incident AF.^23^

Further, in observational studies, Lp(a) has been identified as a risk factor for atrial thrombi in AF patients, but not with AF incidents itself.^24^ However, these studies were restricted by the number of AF cases and were underpowered to detect more miniature Lp(a) effects on AF.^25^, ^26^ Thus, studies addressing the relationship between AF and Lp(a) demonstrated conflicted results.

According to what we know, no systematic review and meta‐analysis have been published in which the association of Lp(a) and AF is thoroughly studied. Aiming to bridge this gap, we performed a systematic review and meta‐analysis to evaluate this hypothesized association.

## 2 METHODS

This meta‐analysis adhered to the Preferred Reporting Items for Systematic Reviews and Meta‐Analyses (PRISMA) statement.^27^ The study was registered with PROSPERO (registration number CRD42023412132). We searched PubMed, Embase, Cochrane Library, Web of Science, MEDLINE, and ScienceDirect to identify all relevant literature from their inception to March 1, 2023. The following search terms were used with no language restrictions: (“lipoprotein(a)” or “Lp(a)” or “Lp[a]”) AND (“atrial fibrillation” or “atrial flutter”). We also analyzed the listed references, included articles from all eligible studies, and manually searched related articles to identify additional potential studies. The initial screening of titles and abstracts was conducted by two independent investigators who retrieved full‐length essays from all likely studies. Afterwards, a screening using the eligibility criteria was conducted, in which studies were only included if they (1) enrolled patients diagnosed with atrial fibrillation; (2) provided an odds ratio (ORs) or hazard ratios (HRs) with 95% confidence interval (CI) of Lp(a) for risk of atrial fibrillation. Studies were excluded if they were reviews, editorials, abstracts, or conference presentations. All decisions regarding eligibility were made according to prespecified selection criteria. We resolved any discrepancies through consensus or discussion with a third investigator.

Two investigators independently extracted relevant elements from each screened article. If available, the following parameters were obtained from each study: first author's name, year of publication, number of participants, demographic information including age and gender, study design, country of origin, Lp(a) levels, and follow‐up periods. The outcomes to be considered in the comprehensive analysis were decided by selecting the studies according to the abovementioned criteria. The Jadad scale for randomized controlled studies was utilized to conduct a study quality assessment.^28^ For nonrandomized controlled studies, two independent investigators assessed study quality using a nine‐item Newcastle‐Ottawa Scale (NOS).^29^ A score of ≥5 was considered high quality. Any discrepancy was resolved by re‐evaluation and consensus among the authors. Statistical analysis was performed using RevMan 5.3 (Cochrane Collaboration) and Stata 14 software (STATA Corporation). ORs or HRs with 95% CIs were used as the summary statistic for dichotomous outcomes. Overall risk estimates for dichotomous data were calculated using the Mantel–Haenszel and inverse‐variance methods. Statistical heterogeneity of all included studies was evaluated by Cochran's *Q* test and *I* ^2^ statistic, where a Q‐statistic *I* ^2^ > 50% or a *p* < .05 suggested high heterogeneity. For cases where *I* ^2^ > 50%, a random‐effect model was used to assess the impact of an intervention. We implemented a fixed‐effect model for cases where *I* ^2^ < 50%. We then conducted sensitivity and subgroup analyses based on country of origin and other factors that may cause heterogeneity. A *p* < .05 was considered statistically significant.

## 3 RESULTS

The literature search identified 672 potentially eligible literature by searching electronic databases. Among them, we excluded 83 studies due to repetitions. Subsequently, 537 articles were considered irrelevant studies by evaluating titles and abstracts. A full text of 52 eligible studies was then reviewed, and 43 records were eliminated since they were reviews, abstracts, letters, and conferences. Therefore, nine studies were included in the final analysis.^11^, ^13^, ^20^, ^25^, ^26^, ^30^, ^31^, ^32^, ^33^ Four were prospective cohort studies^11^, ^23^, ^24^, ^28^ and one was retrospective in design.^31^ Four included studies performed Mendelian randomization (MR) analysis for Lp(a) and AF.^13^, ^20^, ^32^, ^33^ Data on associations between the single‐nucleotide polymorphisms (SNPs) for Lp(a) level and AF were obtained from the online report on the platform of genome‐wide association studies (GWAS). Five studies were from China, and four were from America and Canada. The detailed characteristics of each study are shown in Table 1. All studies included in our meta‐analysis were high quality (Table 2). Four studies have different stratification of the levels of Lp(a).^25^, ^26^, ^30^, ^31^

**Table 1:** Characteristics of included studies.

References	Region	Study design	Sample size	Age	Male (%)	Lp(a) levels (mg/dL)	Follow‐up duration	NOS score
Mora et al.^30^	America	Prospective cohort	23,738	52.6 (48.8, 58.3)	NA	Quintile 1 to 5	Median 16.4 years	9
Li et al.^11^	China	Prospective cohort	679	Mean 70.4 years	394 (58.0)	Atrial fibrillation at admission: 40 ± 20 No atrial fibrillation at admission: 30 ± 20	25.3 ± 0.6 months	9
Aronis et al.^26^	America	Prospective cohort	15,792	62.7 ± 5.6	4346 (27.5)	≤10; >10 to 20; >20 to 30; >30 to 50; >50	13.9 (12.4–14.8 years)	9
Garg et al.^25^	America	Prospective cohort	6593	Mean 62 years	3099 (47)	≤ 10.0; 10.1–29.8; ≥29.9	Median 12.9 years	9
Xia et al.^32^	China	Mendelian randomization (GWAS data sets)	NA	NA	NA	NA	NA	7
Jiang et al.^20^	China	Mendelian randomization (GWAS data sets)	NA	NA	NA	NA	NA	7
Wang et al.^33^	China	Mendelian randomization (GWAS data sets)	NA	NA	NA	NA	NA	7
Mohammadi Shemirani et al.^13^	Canada	Mendelian randomization (GWAS datasets)	NA	NA	NA	NA	NA	7
Tao et al.^31^	China	Retrospective cohort	13,533	Mean 65 years	7185 (53.1)	≤ 8.71; 8.71–16.54; 16.54–32.42; > 32.42	NA	7

Abbreviations: GWAS, genome‐wide association studies; NA, not applicable; NOS, Newcastle‐Ottawa Scale.

**Table 2:** Study quality assessment using the Newcastle‐Ottawa Scale.

	Selection					Outcome			
References	Representativeness of exposed cohort	Selection of nonexposed cohort	Ascertainment of exposure	Outcome of interest absent at start of study	Comparability	Assessment of outcome	Follow‐up long enough for outcome to occur	Adequacy of follow‐up	Total score
Mora et al.^30^	*	*	*	*	* *	*	*	*	9
Li et al.^11^	*	*	*	*	* *	*	*	*	9
Aronis et al.^26^	*	*	*	*	* *	*	*	*	9
Garg et al.^25^	*	*	*	*	* *	*	*	*	9
Xia et al.^32^	*	*	*	*	* *	*	…	…	7
Jiang et al.^20^	*	*	*	*	* *	*	…	…	7
Wang et al.^33^	*	*	*	*	* *	*	…	…	7
Mohammadi Shemirani et al.^13^	*	*	*	*	* *	*	…	…	7
Tao et al.^31^	*	*	*	*	* *	*	…	…	7

*Note*: * indicates one score and … indicates no score.

**Figure 1:**
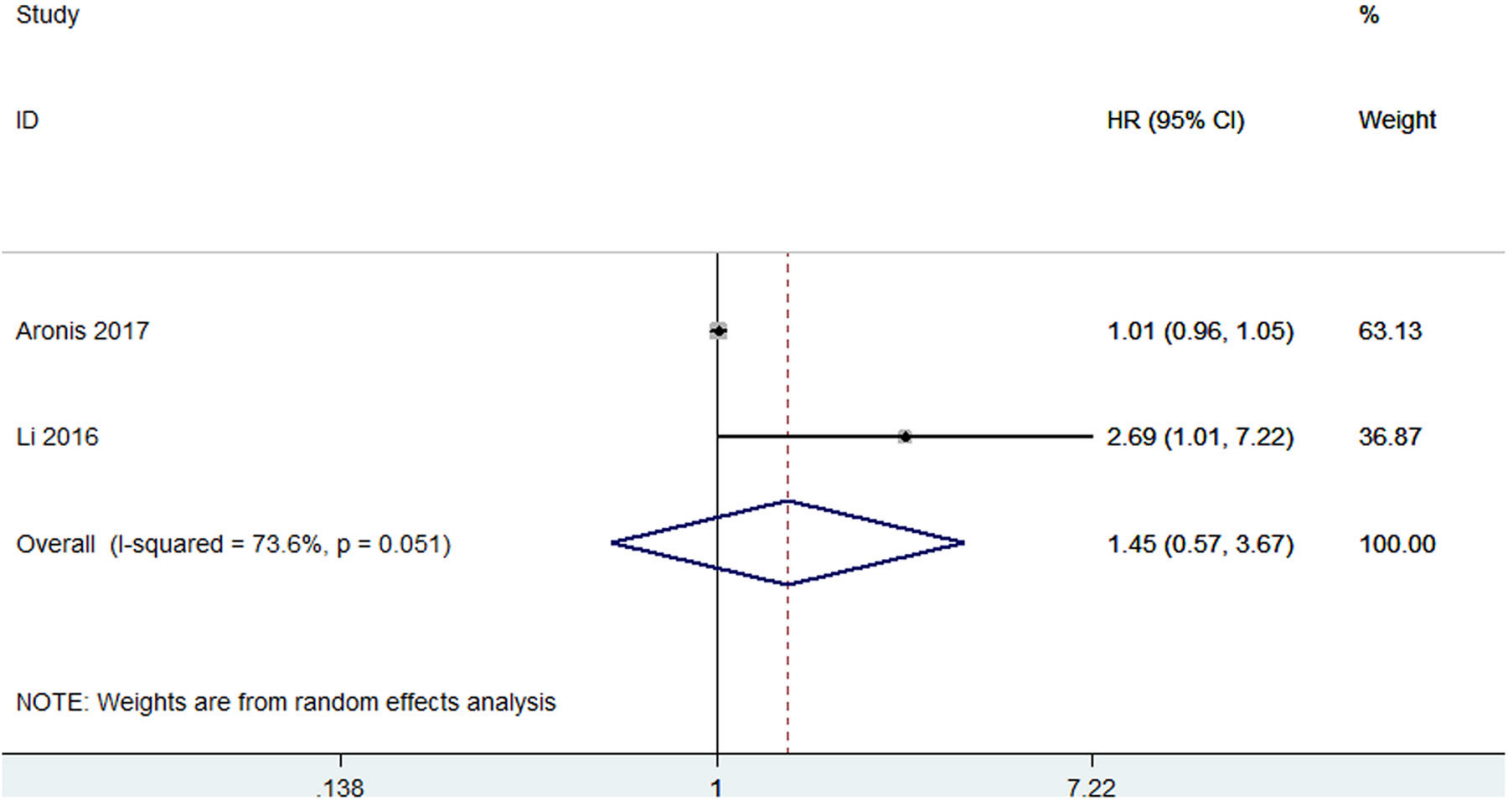
Meta‐analysis of the association between Lp(a) and atrial fibrillation.

**Figure 2:**
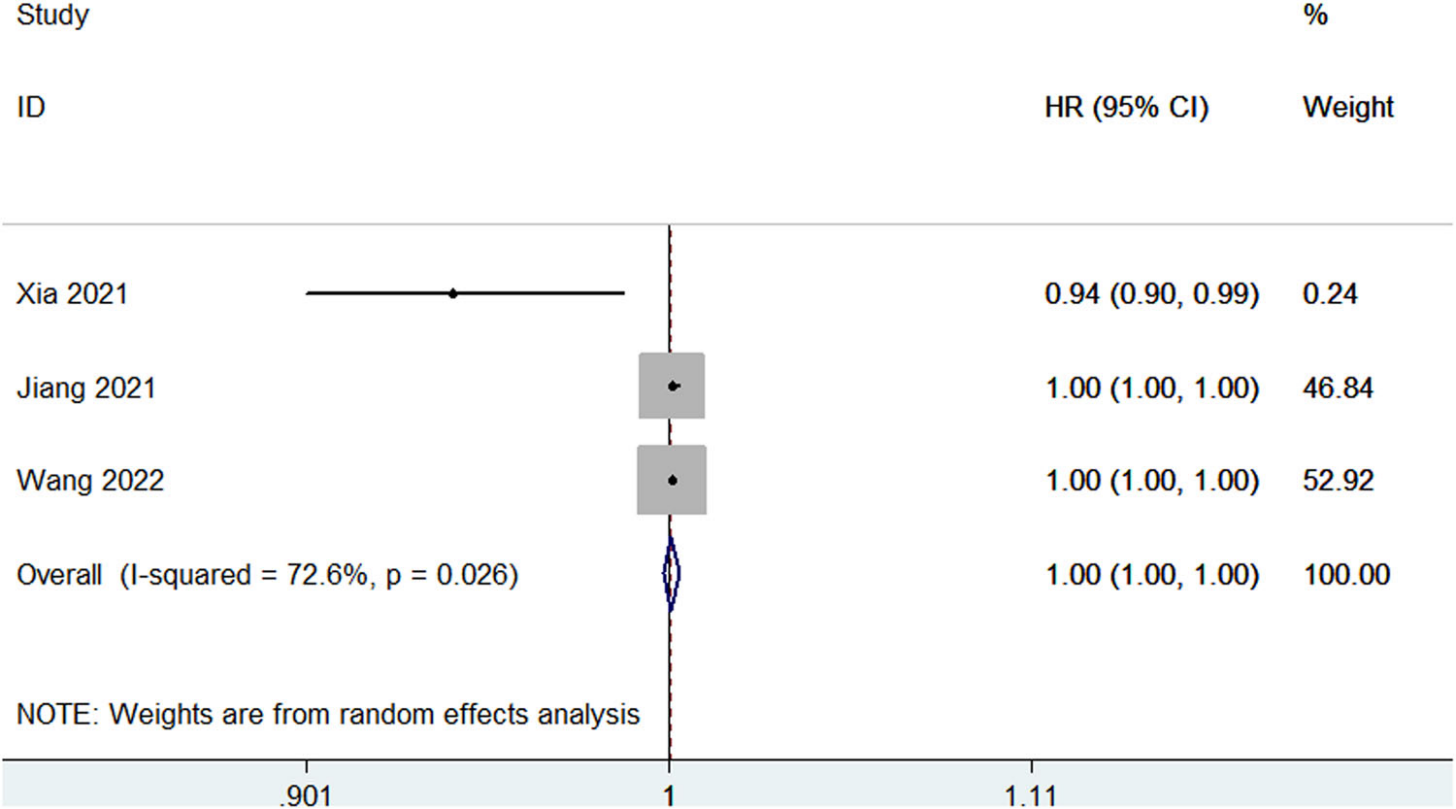
Meta‐analysis of the association between genetically elevated Lp(a) and atrial fibrillation.

This meta‐analysis showed that Lp(a) was not associated with new‐onset AF (HR = 1.45, 95% CI: 0.57–3.67, *p* = .432; *I* ^2^ = 73.6%) (Figure 1). In addition, genetically elevated Lp(a) was not associated with the risk of atrial fibrillation (OR = 1.00, 95% CI: 1.00–1.00, *p* = .461; *I* ^2^ = 72.6%) (Figure 2). Different stratification of Lp(a) levels may have different outcomes. Also, higher Lp(a) levels may be inversely associated with the risk of developing AF compared to those with lower levels.^25^, ^26^, ^31^, ^32^

## 4 DISCUSSION

To the best of our knowledge, the current study is the first systematic review and meta‐analysis that evaluates the association between Lp(a) and AF. Our findings indicate no significant association between Lp(a) and AF incidence.

Some studies have assessed the relationship of AF with Lp(a). For example, Garg and colleagues performed a multiethnic cohort study which eventually included A total of 6593 participants in the final analysis (mean age = 62 years; 53% female; 38% White, 28% African American, 22% Hispanic, and 12% Chinese). The study results showed that an Lp(a) level ≥30 mg/dL was inversely associated with the risk of developing AF compared to those with lower levels.^25^ Another study conducted by Tao and colleagues showed a significant inverse with lower circulating Lp(a) being related to elevated AF incidence. The study also indicated that an Lp(a) level lower than 32.42 mg/dL could be a potential risk factor for AF.^31^ Subsequently, both studies mentioned above suggested that Lp(a) can be used as a helpful marker for AF risk stratification.

Furthermore, other studies addressing the association between Lp(a) and AF incidence have covered the topic from a genetic point of view. Shemirani et al.^13^ conducted a study measuring and genetically predicting Lp(a) levels in 20 432 patients with incident AF in the UK Biobank. Results implicate Lp(a) as a potential causal mediator in the development of AF, demonstrating an Lp(a) effect which extends across myocardial tissues. Besides, Xia et al.^32^ conducted a study proving that genetically elevated Lp(a) was inversely associated with the risk of atrial fibrillation. Overall, epidemiologic and genetic analyses strongly suggest that Lp(a) plays a role in the incident development of AF independent of atherosclerotic cardiovascular disease. These analyses also demonstrate that Lp(a) affects myocardial tissues outside the aortic valve and coronary arteries. This may indicate new disease pathways and pharmacological targets that need further investigation and evaluation.

As previously mentioned, the effects of Lp(a) on incident AF were partly independent of coronary artery disease and atherosclerosis, suggesting that the potential mechanism of Lp(a) on increased AF incidence is different from the known atherosclerotic mechanisms.^13^, ^34^ One possible mechanism is that Lp(a) induces a pro‐inflammatory milieu, which interferes with atrial remodeling and electrical signaling. The preference of oxidized phospholipids to bind to Lp(a) rather than to other LDL particles proves this. These oxidized phospholipids stimulate the production of the inflammatory proteins interleukin‐8 and monocyte chemoattractant proteins. Recent research also reveals that the pathophysiology of Lp(a) depends on this pro‐inflammatory impact.^35^, ^36^, ^37^

Regarding Lp(a) inversed relationship with AF, as Tao et al.^31^ suggested in a large‐scale retrospective cohort study indicated that Lp(a) level lower than 32.42 mg/dL could be a potential risk factor for AF. It was interesting to note that only women showed this association. The findings of the BiomarCaRE Consortium similarly demonstrated that total cholesterol and other proatherogenic lipoproteins, such as Lp(a), are protective factors against AF, particularly in women.^38^ Similar findings from this study indicated that women's cholesterol levels are considerably higher than men's.^39^ However, according to the Women's Health Study in Switzerland, small LDL particles with low cholesterol, rather than large LDL particles with high cholesterol like Lp(a), are responsible for the negative connection with AF.^30^ The mechanism for such an effect is not fully explained; Thus, well‐designed prospective clinical trials will determine whether sex influences the association between Lp(a) and AF.

AF is a growing public health problem, with an expected prevalence increase in the coming years. Lp(a) may have a causal relationship with incidence AF, independent of its effects on coronary artery disease and atherosclerosis. This connection between Lp(a) and AF may also help to explain how Lp(a) mediates its harmful effects on the nervous system by increasing thromboembolic events, as cerebrovascular diseases are highly related to raised Lp(a) levels. The precise molecular pathways by which Lp(a) influences AF risk and atrial remodeling are still unknown, thus, need to be further studied. Conducting studies that address the relationship or the exact mechanism of LP(a) levels and incident AF is of massive importance. Through utilizing suggested mechanisms, scientists can provide prevention and treatment measures that may help reduce the prevalence of AF and its complications, such as heart failure and stroke. This will significantly impact how we perceive the most common type of arrhythmia worldwide, leading to better prevention and treatment strategies. In general, strategies to lower Lp(a) should be prioritized to decrease the morbidity and mortality of the elderly population, thus reducing the public pardon of AF.

Our study has several limitations, so our results should be interpreted cautiously. Our sample size was small, with limited included articles in the meta‐analysis, potentially creating the risk of publication bias. Furthermore, because some studies have different stratification of Lp(a) level, the meta‐analysis of Lp(a) prediction for AF was only in several studies, thus having even smaller sample sizes subgroup analyses based on stratification of Lp(a) levels and ethnicity were not able to be conducted. The heterogeneity across the included studies was significant. Our results emphasize the need for randomized clinical trials on the effect of lowering Lp(a) levels to prevent AF. Despite these limitations, this study is the first systematic review and meta‐analysis to investigate the association between Lp(a) and new‐onset AF. Additional research is needed to provide a larger sample size and better understand Lp(a) stratification for AF and the possible inverse association between Lp(a) and AF.

## 5 CONCLUSION

Lp(a) levels were not associated with incident AF. Further research is needed to identify the mechanism underlying these results and better understand Lp(a) stratification for AF and the possible inverse association between Lp(a) and AF.

## AUTHOR CONTRIBUTIONS

*Conceive, design, and critical revision of the manuscript for important intellectual conten*t: Mingyang Yang. *Data collection, analysis, manuscript drafting*: Basma Nasr. *Data collection, analysis, manuscript drafting*: Junzhao Liu. *Quality assessment of the included studie*s: Yu Du. *Quality assessment of the included studies*: Jiayin Yang. All authors reviewed the manuscript.

## CONFLICT OF INTEREST STATEMENT

The authors declare no conflict of interest.

## Data Availability

The data that support the findings of this study are available from the corresponding author upon reasonable request.
